# Atomistic Insight into the Hydration States of Layered Double Hydroxides

**DOI:** 10.1021/acsomega.2c01115

**Published:** 2022-04-02

**Authors:** Xuejiao Li, Tim Würger, Christian Feiler, Robert H. Meißner, Maria Serdechnova, Carsten Blawert, Mikhail L. Zheludkevich

**Affiliations:** † Institute of Surface Science, Helmholtz-Zentrum Hereon, Geesthacht 21502, Germany; ‡ Institute of Polymers and Composites, Hamburg University of Technology, Hamburg 21073, Germany; ¶ Institute for Materials Science, Faculty of Engineering, Kiel University, Kiel 24103, Germany

## Abstract

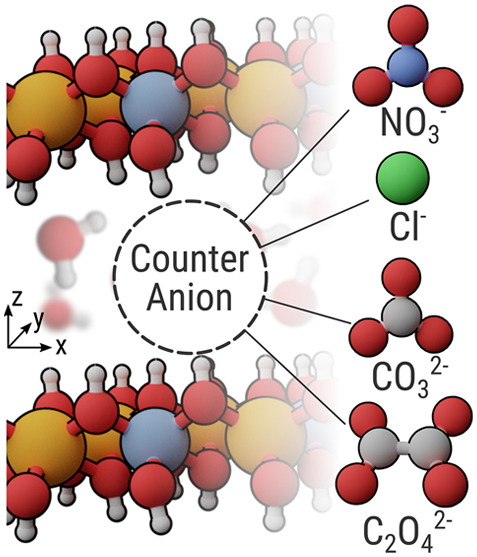

Effective protective coatings are an essential component of lightweight engineering materials in a large variety of applications as they ensure structural integrity of the base material throughout its whole service life. Layered double hydroxides (LDHs) loaded with corrosion inhibitors depict a promising approach to realize an active corrosion protection for aluminum and magnesium. In this work, we employed a combination of density functional theory and molecular dynamics simulations to gain a deeper understanding of the influence of intercalated water content on the structure, the stability, and the anion-exchange capacity of four different LDH systems containing either nitrate, carbonate, or oxalate as potential corrosion inhibiting agents or chloride as a corrosion initiator. To quantify the structural change, we studied the atom density distribution, radial distribution function, and orientation of the intercalated anions. Additionally, we determined the stability of the LDH systems by calculating their respective hydration energies, hydrogen-bonded network connected to the intercalated water molecules, as well as the self-diffusion coefficients of the intercalated anions to provide an estimate for the probability of their release after intercalation. The obtained computational results suggest that the hydration state of LDHs has a significant effect on their key properties like interlayer spacing and self-diffusion coefficients of the intercalated anions. Furthermore, we conclude from our simulation results that a high self-diffusion coefficient which is linked to the mobility of the intercalated anions is vital for its release via an anion-exchange mechanism and to subsequently mitigate corrosion reactions. Furthermore, the presented theoretical study provides a robust force field for the computer-assisted design of further LDH-based active anticorrosion coatings.

## Introduction

Transportation applications are one of the major driving forces behind the increasing impact of global warming.^1^ Fortunately, the application of light metal alloys, especially in transportation industries, substantially decreases the consumption of fossil fuels and subsequently combats and mitigates ongoing climate change.^2^ Among the most promising materials are aluminum (Al) and magnesium (Mg) alloys which exhibit exceptional strength to weight ratios.^2,3^ However, effective protection strategies are required due to their comparably low corrosion resistance. For this reason, organic coatings are widely employed to protect light metal alloys from corrosion and to preserve the integrity of the structural material.^4−6^ The protection efficiency of such coatings can decrease significantly after formation of defects caused by aging or fatigue.^6^ To overcome this issue, different self-healing approaches were proposed over the past decade.^7^ Such active corrosion protection can be achieved by encapsulating corrosion-inhibiting agents in the coating, which are released by external stimuli (e.g., chemical or mechanical).^8,9^ This release is, however, noncontrollable since all of the corrosion-inhibiting agents can be released rapidly after a defect occurs.^6^

A versatile approach to realize a controllable active corrosion protection scheme is the inclusion of layered double hydroxides (LDHs) intercalated with divanadate or decavanadate anions. Coatings of this type provide good long-term corrosion protection for aluminum and magnesium alloys, indicating that LDHs intercalated with corrosion inhibiting agents are promising candidates to replace less sustainable and toxic corrosion protection strategies based on hexavalent chromates.^6,8−12^ An LDH is an inorganic sheetlike clay exhibiting a brucite structure in its pure Mg(OH)_2_ form. Its composition is highly versatile since the magnesium atoms in the sheet can be substituted with different metal ion such as divalent (e.g., Zn^2+^) or trivalent (e.g., Al^3+^).^13^ The latter causes a positive net charge in the sheets,^13−15^ which can be balanced by intercalation of anions that may simultaneously act as potential corrosion inhibiting agents. The general formula of the LDH family is [M_1–*x*_^2+^M_*x*_^3+^(OH)_2_]^*x*+^A_*x*/*n*_^*n*–^·*m*H_2_O, where M^2+^ and M^3+^ depict divalent and trivalent cations, respectively, A^*n*–^ an intercalated anion, and *x* a molar ratio M^3+^/(M^2+^ + M^3+^).^4,15−17^ A typical composition in corrosion protection applications is a combination of the divalent cations Mg^2+^ or Zn^2+^ with Al^3+^. Apart from the previously mentioned vanadate anions, inorganic anions like phosphate,^12^ tungstate,^18^ and molybdate^19^ as well as organic anions like quinaldate,^20^ laurate,^21^ and 2-mercaptobenzothiazolate^20^ have also been studied after intercalation in an LDH with the aim of augmenting the anticorrosion property of the resulting organic coating. However, there is an essentially infinite number of potential inhibiting agents that may be incorporated into the intergallery spaces of an LDH.^22^ Hence, the use of computational techniques is indispensable for a deeper understanding of LDH-based corrosion protection schemes. The key step for the corrosion protection achieved by LDHs intercalated with corrosion inhibitors is the release of the intercalated inhibitor and the simultaneous uptake of aggressive anions such as Cl^–^ that initiate corrosion of Mg-based materials through an anion-exchange mechanism.^6,8^ On the basis of an experimental study, Hou et al. pointed out that the hydration state of the LDH can be linked to its anion-exchange behavior.^23^ The affinity of different anions to a metal hydroxide layer with an identical Mg^2+^/Al^3+^ ratio has been studied from both experimental^24^ and theoretical^25^ perspectives. It is noteworthy that both resulted in an identical affinity order: NO_3_^–^ < Br^–^ < Cl^–^ < F^–^ < OH^–^ < SO_4_^2–^ < CO_3_^2–^. As NO_3_^–^ has the lowest affinity and can be easily replaced by other anions, LDHs intercalated with NO_3_^–^ are widely used as precursor by many research groups to obtain novel LDH host–guest systems by anion exchange between NO_3_^–^ and a suitable anion of interest.^26,27^

Although LDHs have been extensively characterized by experimental investigations,^6,12,23,28^ it remains highly challenging to obtain a comprehensive insight into the LDH interlayer from purely experimental investigations. Fortunately, complementary theoretical investigations can assist in unraveling the chemistry within the LDH intergalleries. For this sake, density functional theory (DFT) has been extensively applied to study the chemically induced structural change^29,30^ and the electronic properties^31−33^ of LDH-type structures. However, it is computationally expensive to study the LDH structure with DFT on a large scale since the respective simulations are afflicted with long computational time for systems including several thousands atoms, required to investigate a multitude of LDH properties while avoiding finite size effects.^34,35^ Nevertheless, the results of these comparably expensive simulations provide starting geometries and atomic charges of the included atoms for other methods, e.g., molecular dynamics (MD), that are more suited to investigate large systems on acceptable time scales.^15,34^ Concomitantly, MD simulations have been widely applied to gain more insights in LDH structures,^34,36−41^ interactions of the intercalated compounds and metal hydroxide layer,^34,36−38,40,42,43^ and dynamic properties of the intercalated compounds^40,42,44^ at considerably lower computational costs.

**Figure 1:**
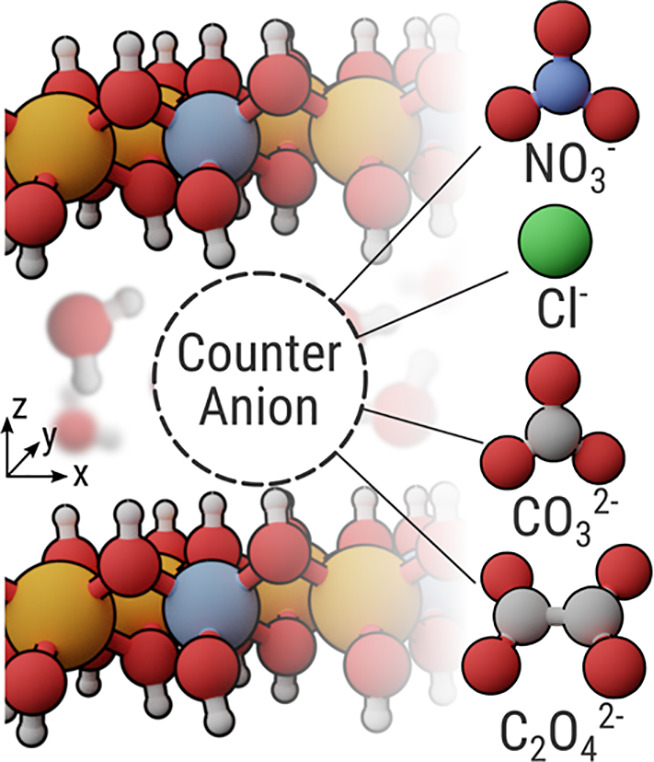
Schematic representation of a layered double hydroxide system with the counteranions investigated in this study.

The influence of the hydration state on LDHs intercalated with amino acids has been widely studied to explore the origin of life^34,45^ and to study drug and gene delivery.^44^ Some of these studies prove that hydration is a crucial aspect in determining the stability of LDHs and is correlated to the anion-exchange behavior. In this work, we therefore investigate the influence of hydration states on the Al-Mg-LDH (for simplicity referred to as LDH in the following paragraphs). This composition of LDH was selected since it has been applied in corrosion protection for Al and Mg alloys.^20,27^ We use four different LDH systems as the basis for our study, intercalated with nitrate (NO_3_^–^), carbonate (CO_3_^2–^), oxalate (C_2_O_4_^2–^), and chloride (Cl^–^), respectively (see Figure 1). The latter is of great interest as Cl^–^ is an aggressive species that initiates corrosion progression in Mg-based materials and may act as a chemical trigger for the ion exchange. As a result, the corrosion progression can be mitigated by exchange of Cl^–^ with an intercalated corrosion inhibiting agent. The inhibitor selected for this work is oxalate (C_2_O_4_^2–^) since it has shown an exceptional corrosion inhibiting effect for a large variety of different magnesium alloys.^22^ Furthermore, it was already demonstrated that oxalate can be intercalated in an LDH, and it thus depicts a promising starting point for the realization of an effective anticorrosion coating based on LDHs.^6^ Although LDHs intercalated with nitrate (NO_3_^–^), chloride (Cl^–^) and carbonate (CO_3_^2–^) have been studied in previous simulations, the details on the influence of hydration state are still missing. Moreover, there is limited information available in the literature on the LDH intercalated with oxalate (C_2_O_4_^2–^). In this study, a combination of DFT calculations and MD simulations was carried out to gain a deeper understanding of the four LDH systems in different hydration states and reveal a possible link between the hydration state and anion-exchange capacity.

## Results and Discussion

Tavares et al.^46^ pointed out that hydration can induce changes in the electronic structure of an LDH, which in turn could affect the partial charges of the atoms in the LDH. Moreover, the partial charge parameters of the force field can greatly affect the calculation of the electrostatic interactions and heavily influence the outcome of MD simulations and in particular the stability of LDH.^15^ Hence, it is essential to ensure that the charge values that are used as basis to parametrize the employed force field are reasonable, reliable, and ideally also compatible with other commonly used force fields. For this sake, the partial charges were calculated at different hydration states for the atoms in the metal hydroxide (MOH) layer as well as for the intercalated molecules (see Table 1) in each unit cell using DFT calculations in combination with the atomic population analysis method DDEC6 (for details see the Methods). The results show that the influence of the water content on the partial charges can be neglected as they are unaffected by the number of intercalated water molecules (see Figure S1). Note that the partial charges of different types of atoms were averaged over different hydration states. After that, the partial charges of the atoms in the MOH layer were averaged over the four LDH systems and partial charges of different intercalated anions were streamlined to approximate, homogenize, and account for the different oxidation states in the system. For the intercalated water molecules, we employed the partial charges from the SPC/E model.^47^ Finally, the values listed in Table 1 were subsequently used as the basis for the MD simulations.

**Table 1:** DDEC6-Derived Partial Charges (*q*, in au) of All Types of Atoms Used in MD Simulations for LDH-NO_3_^–^, LDH-Cl^–^, LDH-CO_3_^2–^, and LDH-C_2_O_4_^2–^

	element	LDH-NO_3_^–^	LDH-Cl^–^	LDH-CO_3_^2–^	LDH-C_2_O_4_^2–^
MOH layer	Al	1.7700			
	Mg	1.4400			
	O(LDH)	–1.0800			
	H(LDH)	0.4000			
Intergallery	O(NO_3_^–^)	–0.4300	-	-	-
	N(NO_3_^–^)	0.7200	-	-	-
	Cl^–^	-	–0.5700	-	-
	O(CO_3_^2–^)	-	-	–0.6600	-
	C(CO_3_^2–^)	-	-	0.8400	-
	O(C_2_O_4_^2–^)	-	-	-	–0.5340
	C(C_2_O_4_^2–^)	-	-	-	0.4980
	O(H_2_O)	–0.8476	–0.8476	–0.8476	–0.8476
	H(H_2_O)	0.4238	0.4238	0.4238	0.4238

**Figure 2:**
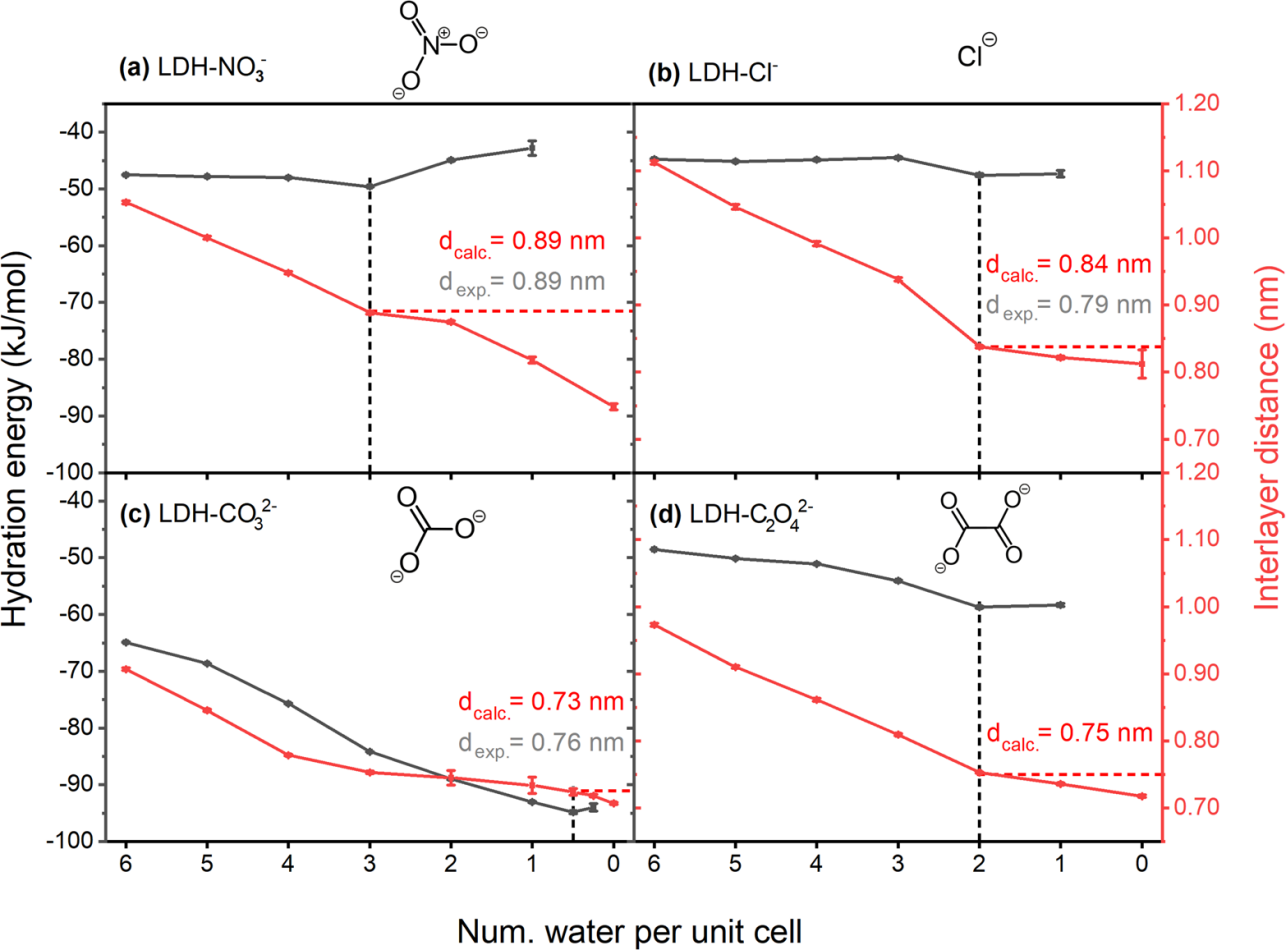
Hydration energy (black) and interlayer distance (red) as a function of the water content. The dotted black lines are used as guide for the eye to highlight the most stable hydration states for each of the investigated LDH systems (a) LDH-NO_3_^–^, (b) LDH-Cl^–^, (c) LDH-CO_3_^2–^, and (d) LDH-C_2_O_4_^2–^. In a similar fashion, the dotted red lines were included to mark the corresponding interlayer distance. The values of the interlayer distance obtained from simulations (*d*_calc_) are written in red, while the corresponding experimental reference (*d*_exp_) is shown in gray. The experimental reference for the LDH-C_2_O_4_^2–^ system cannot be provided in this work as the exchange procedure needs to be further optimized.

The interlayer distance of each type of LDH decreases with lower water content as displayed by the red lines in Figure 2. The black lines illustrate the hydration energy of the LDHs with respect to different water contents. The most stable hydration state is defined by the water content corresponding to the minimum hydration energy for LDH-NO_3_^–^ (three H_2_O per unit cell) or the water content initiating a plateau for LDH-Cl^–^ (two H_2_O per unit cell), LDH-CO_3_^2–^ (half H_2_O per unit cell, which equals one H_2_O per two unit cells), and LDH-C_2_O_4_^2–^ (two H_2_O per unit cell). It is noteworthy that there is no further decrease in hydration energy when the number of water molecules per unit cell is below one for the LDH-Cl^–^ and LDH-C_2_O_4_^2–^ systems. For the LDH-CO_3_^2–^ system, the hydration energy is clearly lower than for the other three systems investigated in this work, indicating the extremely high stability of the intercalated CO_3_^2–^ as mentioned by Sasai et al.^48^ Its stable hydration state occurs for a half water molecule per unit cell, which was obtained by removing half of the total amount of water at the state with one water per unit cell. The interlayer distance at the most stable hydration state is the same as the experimental reference of 0.89 nm for the LDH-NO_3_^–^ system.^49^ For the LDH-Cl^–^ system, the interlayer distance is 0.84 nm, which is slightly higher than the experimentally determined value of 0.79 nm.^50^ The interlayer distance of LDH-CO_3_^2–^ is 0.73 nm at its most stable hydration state, which is slightly lower than the experimental value 0.76 nm. The interlayer distance of LDH-C_2_O_4_^2–^ is 0.75 nm, which is smaller than that of the LDH-Cl^–^ system and very close to the experimentally known interlayer distance of the LDH-CO_3_^2–^ of 0.76 nm.

To elucidate the orientation and position of the intercalated anions relative to the metal hydroxide layer, the number density of the principal atoms that are listed in Table 1 was investigated along the *z*-direction at the most stable hydration state (denoted as stable hydration state) and the higher hydration state next to the most stable hydration state (named high hydration state) for the four systems investigated in this work.

**Figure 3:**
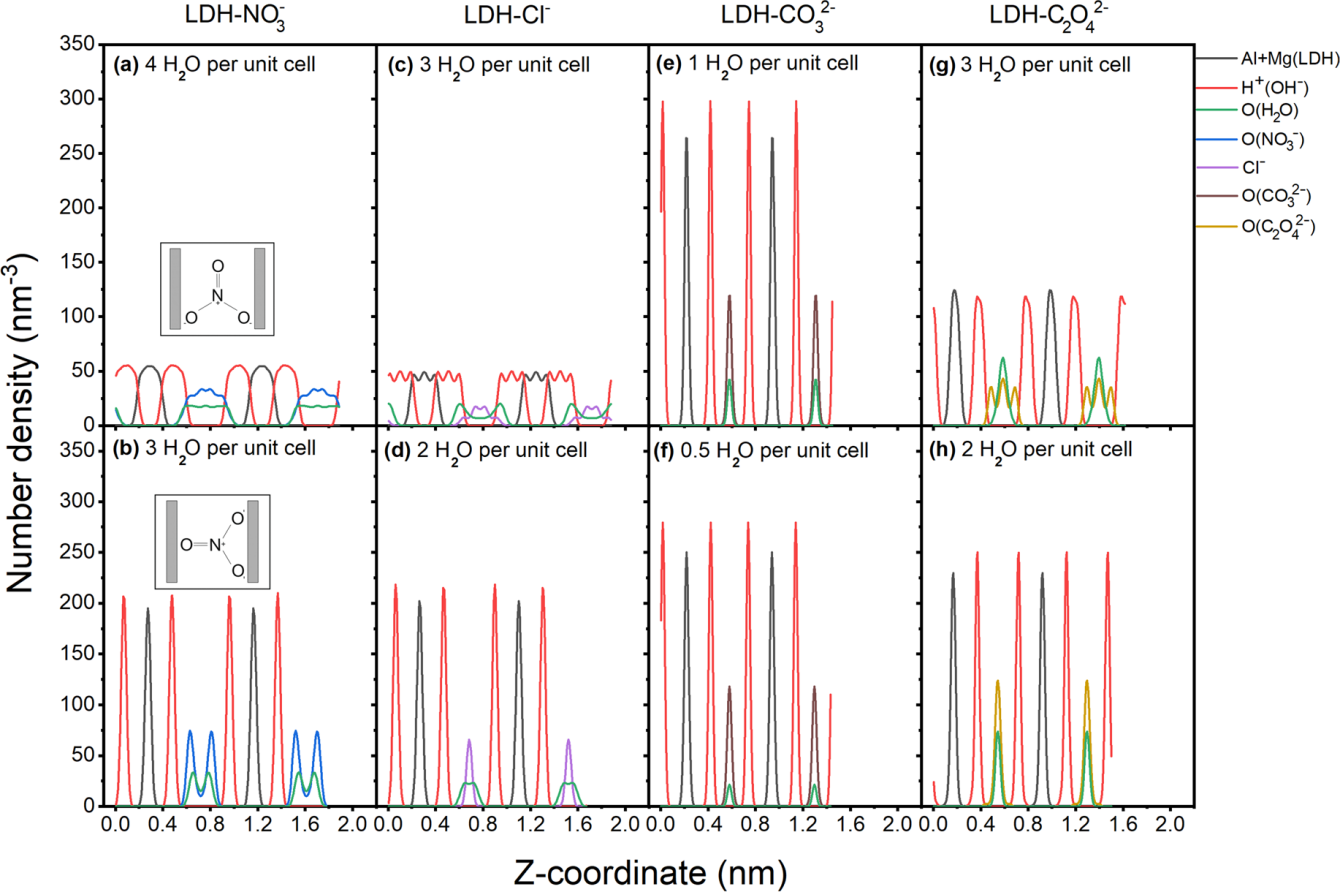
Number density of different types of atoms along the *z*-direction for LDH-NO_3_^–^: (a) high hydration state, four H_2_O per unit cell; (b) stable hydration state, three H_2_O per unit cell. The inserted illustrations are two schematic diagrams of how one NO_3_^–^ can be intercalated between two metal hydroxide layers, represented by gray bars. For LDH-Cl^–^: (c) high hydration state, three H_2_O per unit cell; (d) stable hydration state, two H_2_O per unit cell. For LDH-CO_3_^2–^: (e) high hydration state, one H_2_O per unit cell; (f) stable hydration state, half H_2_O per unit cell. For LDH-C_2_O_4_^2–^: (g) high hydration state, three H_2_O per unit cell; (h) stable hydration state, two H_2_O per unit cell. Black depicts aluminum and magnesium, red the hydrogen atoms of the hydroxide group in the MOH layer, green the oxygen of the intercalated water molecules, blue for the oxygen atoms of nitrate, purple for chloride, brown for oxygen atoms in carbonate, and ochre for oxygen atoms in oxalate.

**Figure 4:**
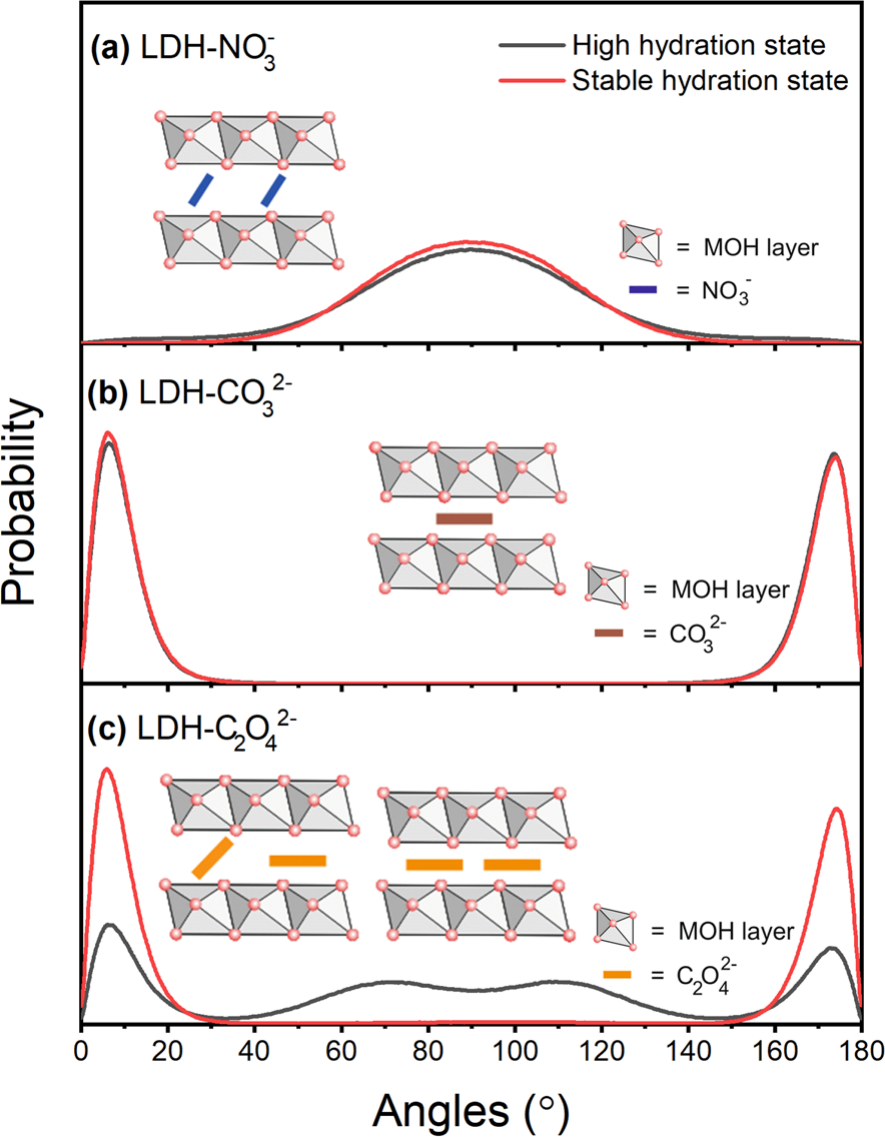
Angle distribution between anion plane and the metal hydroxide (MOH) layer (the definition of the anion plane for NO_3_^–^, CO_3_^2–^, and C_2_O_4_^2–^ is provided in Figure S2). (a) Angle distribution between NO_3_^–^ plane and MOH layer at high and stable hydration states. (b) Angle distribution between CO_3_^2–^ plane and MOH layer at high and stable hydration states. (c) Angle distribution between C_2_O_4_^2–^ plane and MOH layer for high and stable hydration states. The inserted illustrations in (a–c) show the schematic diagrams at the two hydration states for the respective system, where the left section depicts the high and the right section the stable hydration state in (c). Black lines depict the high and red lines the stable hydration state.

The number densities of the atoms belonging to the metal hydroxide layers (Al, Mg, H (OH^–^)) of LDH-CO_3_^2–^ and LDH-C_2_O_4_^2–^ are analogous for the high and stable hydration states as shown in Figure 3e–h. This indicates that the metal hydroxide layers are stable in both states and are not affected by the changes in intercalated water content for these two types of LDHs. The metal hydroxide layers of LDH-NO_3_^–^ and LDH-Cl^–^ are, however, less stable at the high hydration state compared to the stable hydration state, resulting in the broader peaks in Figure 3a and the wavelike pattern as shown in Figure 3c. Both the peak broadening and the wavelike pattern can be attributed to the condition that the atoms in the metal hydroxide layers are not perfectly in-plane at the high hydration state. This peak-broadening and wavelike pattern disappear at the stable hydration state. Additionally, the distribution of O (from H_2_O) at a high hydration state in Figure 3c is different from the distribution at a stable hydration state in Figure 3d. At a high hydration state, there are two layers of water formed in the interlayer region, and these two layers of water are located next to the metal hydroxide layers. The two layers of water merge into one layer at the stable hydration state in Figure 3d. However, there is no apparent change for the distribution of Cl^–^ at both high and stable hydration states, as shown in Figure 3c,d. Additionally, the distribution of O (from both CO_3_^2–^ and H_2_O) is analogous for the two hydration states in the LDH-CO_3_^2–^ system (Figure 3e,f). For LDH-NO_3_^–^, there are two distinct O peaks of NO_3_^–^ and H_2_O at the stable hydration state (Figure 3b), respectively, indicating that most of the O atoms (of both NO_3_^–^ and H_2_O) are close to the metal hydroxide layers. In contrast, the distribution of O (for both NO_3_^–^ and H_2_O) at the high hydration state is broad over the entire interlayer region (Figure 3a). The difference of the O distribution (of NO_3_^–^) between stable and high hydration states can partially be attributed to the rotation of the trigonal planar structure of the NO_3_^–^ anion, as shown in the inlays in Figure 3a,b. This rotation leads to a smaller interlayer distance and a stronger interaction between the intercalated compounds and the metal hydroxide layers at the stable hydration state. For LDH-C_2_O_4_^2–^, there are three O (C_2_O_4_^2–^) peaks at a high hydration state (Figure 3g), which convert into one O (C_2_O_4_^2–^) peak at a stable hydration state (Figure 3h), indicating that the C_2_O_4_^2–^ plane is first tilted to the metal hydroxide layers before shifting to be parallel, respectively (see Figure 4).

The angle distributions between the NO_3_^–^ plane and the metal hydroxide layer at the high and stable hydration states are similar, as shown in Figure 4a, which indicates that most of the NO_3_^–^ anions are tilted by ∼70° relative to the metal hydroxide layer at both states. This observation is in good agreement with the results from Marappa et al.^51^ and Perez-Sanchez et al.^15^ For the LDH-CO_3_^2–^ system, all CO_3_^2–^ anions are parallel to the metal hydroxide layer at both hydration states (see Figure 4b). Contrary to this, the orientation of the anion in the LDH-C_2_O_4_^2–^ system is strongly affected by the change in hydration state, as shown in Figure 4c. At the high hydration state, over 50% of the intercalated C_2_O_4_^2–^ anions are tilted with respect to the metal hydroxide layer while the anion plane of some C_2_O_4_^2–^ is in parallel to the metal hydroxide layer. At the stable hydration state, all C_2_O_4_^2–^ anions adopt a parallel orientation relative to the metal hydroxide layer, indicating that the orientation of the intercalated C_2_O_4_^2–^ is strongly dependent on the number of present water molecules in the interlayer. This result is corroborated by the behavior of intercalated CO_3_^2–^, which is also observed to be orientated in parallel to the metal hydroxide layer.

**Figure 5:**
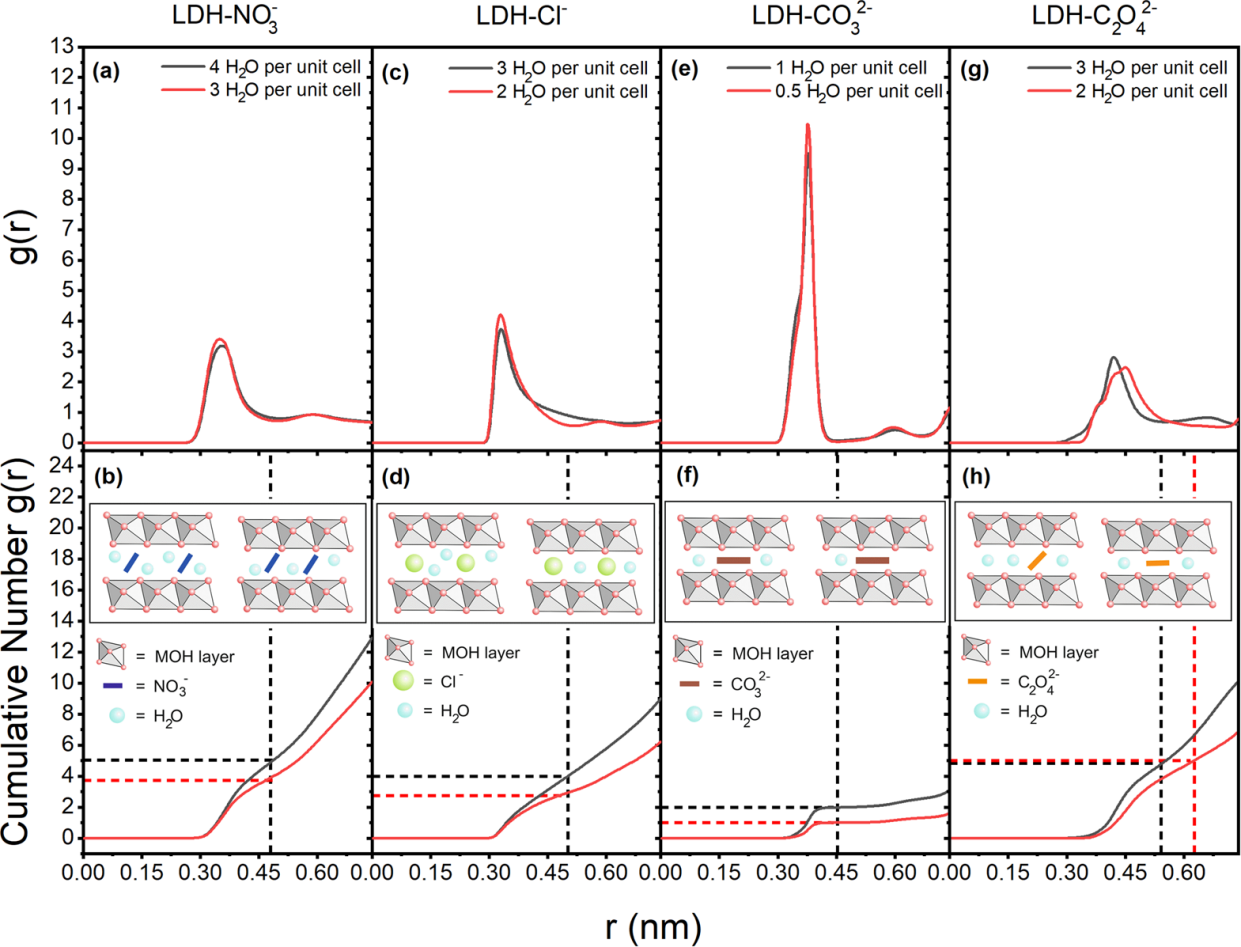
Radial distribution functions *g*(*r*) of water molecules around the intercalated anions and their respective cumulative numbers for the four types of LDHs at high hydration state (in black continuous lines) and stable hydration state (in red continuous lines). For LDH-NO_3_^–^, (a) *g*(*r*) and (b) cumulative number for four and three H_2_O per unit cell. For LDH-Cl^–^, (c) *g*(*r*) and (d) cumulative number for three and two H_2_O per unit cell. For LDH-CO_3_^2–^, (e) *g*(*r*) and (f) cumulative number at one and a half H_2_O per unit cell. For LDH-C_2_O_4_^2–^, (g) *g*(*r*) and (h) cumulative number at three and two H_2_O per unit cell. The insets in (b), (d), (f), and (h) show the schematic diagrams for the four types of LDHs at the two hydration states, with the left section showing the high and the right section the stable hydration state. The vertical dashed lines mark the cutoff distances for the first peak of *g*(*r*) and the cutoffs for calculating the cumulative number of *g*(*r*). The horizontal dashed lines were included to determine the number of coordinated water molecules around the anions at different states.

The radial distribution functions (*g*(*r*)) of the water molecules around the anions were calculated to elucidate the relative position differences between the water molecules and anions at the high and stable hydration states. From the cumulative number of *g*(*r*), one can infer the coordination number of water molecules around the intercalated anions. For LDH-NO_3_^–^, LDH-Cl^–^, and LDH-CO_3_^2–^ in Figure 5a,c,e, respectively, the peak of *g*(*r*) appears at the same distance for both states. The cumulative number calculations in Figure 5b,d,f show that for these three types of LDHs the coordination number of water around NO_3_^–^, Cl^–^, and CO_3_^2–^ is proportional to the amount of intercalated water molecules. When the amount of intercalated water decreases from four H_2_O to three H_2_O per unit cell for LDH-NO_3_^–^, the cumulative number decreases proportionally from 4.9 to 3.7. For LDH-Cl^–^, the cumulative number changes from 4 to 2.7 when the amount of intercalated water is reduced from three H_2_O to two H_2_O per unit cell. The cumulative number of water around the CO_3_^2–^ anions is halved from 2 to 1 after the removal of half of the total intercalated water, moving from the high hydration state to the stable state. The change in the number of coordinated water molecules around the anions for the different hydration states of LDH-NO_3_^–^, LDH-Cl^–^, and LDH-CO_3_^2–^ can be explained by the ionic radii of the involved species. The tilted NO_3_^–^ (179 pm),^52^ Cl^–^ (172 pm),^52^ and CO_3_^2–^ (178 pm)^52^ all exhibit a larger ionic radius than the water molecules (138 pm)^53^ and determine the interlayer spacing of the LDH. Hence, the intercalated water molecules mainly fill the empty space as shown in the respective inlays in Figures 5b,d,f, explaining the correlation between the coordination number and the amount of intercalated water.

For LDH-C_2_O_4_^2–^ (the ionic radius of C_2_O_4_^2–^ is 200 pm^54^), the *g*(*r*) peaks of the two hydration states display slightly different shapes (see Figure 5g). There is a noticeable peak shift of the *g*(*r*) for the stable hydration state (two water molecules) compared to the higher hydration state (three water molecules), as illustrated in Figure 5g. Moreover, the cumulative numbers of *g*(*r*) (Figure 5h) at these two states are close to each other, which varies from the proportional behavior observed in the other three systems. These are related to the angle distribution change as illustrated in Figure 4c. After the water content decreased from three H_2_O to two H_2_O per unit cell, all C_2_O_4_^2–^ anions are orientated parallel with respect to the metal hydroxide layer. Naturally, this implies a rearrangement of the intercalated water molecules that surround the oxalate (see Figure 3h) and a smaller interlayer distance. Since the *g*(*r*) calculation is more constrained in the *z*-dimension at the stable hydration state, the corresponding *g*(*r*) peak is shifted to a higher distance value. This also results in a larger cutoff distance for calculating the cumulative numbers, thus leading to a similar number of water molecules coordinated to the C_2_O_4_^2–^ anion at these two hydration states. Therefore, the number of coordinated water molecules around C_2_O_4_^2–^ is not proportional to the amount of intercalated water molecules, although the ionic radius of C_2_O_4_^2–^ is also larger than that of the water molecules.

**Figure 6:**
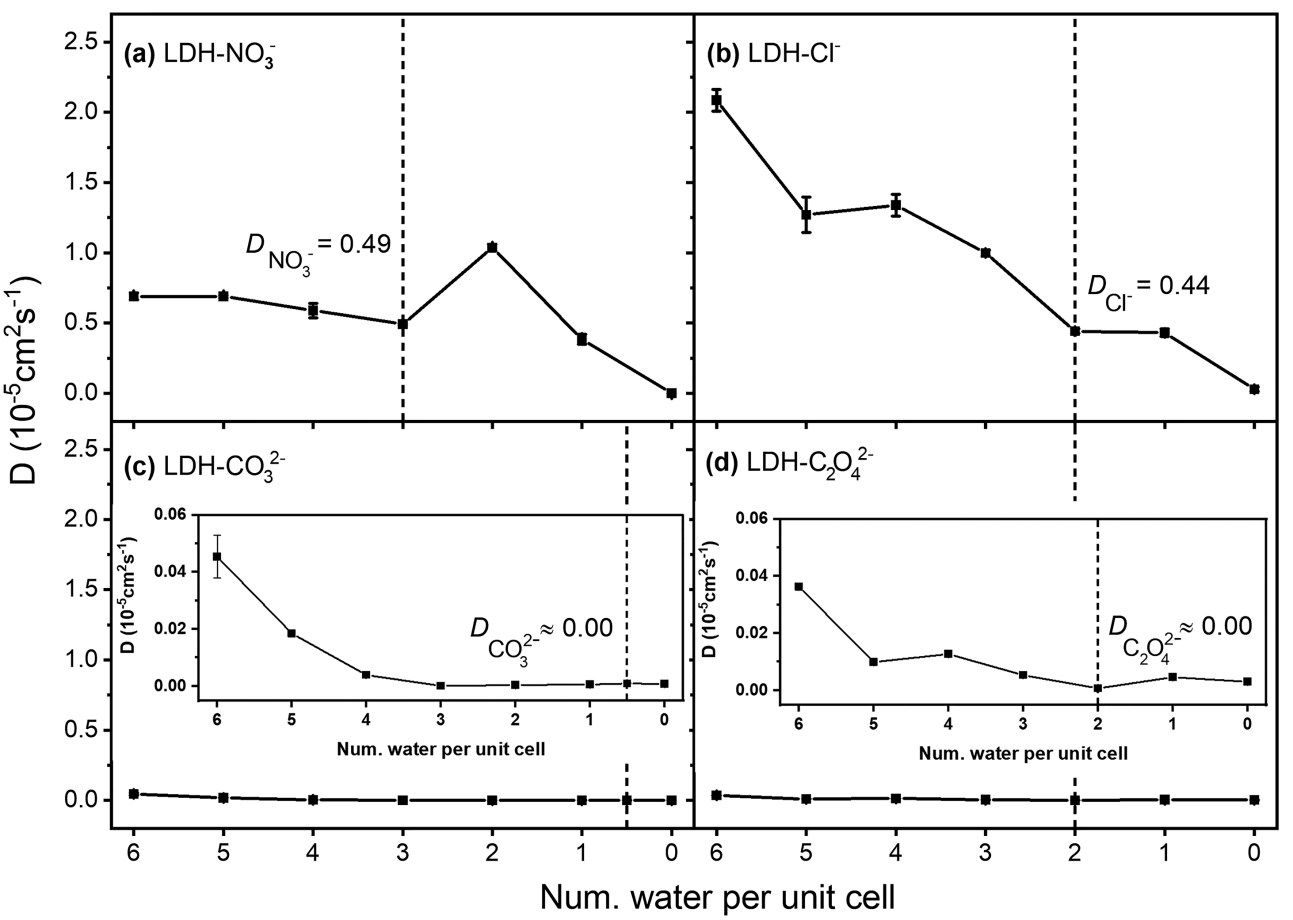
Self-diffusion coefficient (*D*) at different hydration states in *x*- and *y*-direction, calculated for (a) NO_3_^–^, (b) Cl^–^, (c) CO_3_^2–^, and (d) C_2_O_4_^2–^. The inlays in (c) and (d) show the enlarged view in the *y*-direction of (c) and (d). The dotted vertical lines highlight the stable hydration states for each of the investigated LDH systems.

The self-diffusion coefficient (*D*) of the intercalated anions was calculated based on their mean squared displacement (MSD) values (see Figure S3) to determine the stability of the LDH. The simulations indicate that the self-diffusion coefficient of an intercalated anion generally decreases upon presence of less water molecules in the interlayer region. An exception to this observation is the LDH-NO_3_^–^ system. Here, the self-diffusion coefficient of NO_3_^–^ increases when the amount of intercalated water is lowered from three H_2_O (stable state) to two H_2_O per unit cell (Figure 6a). This can be attributed to the implied subtle change in the interlayer distance (see Figure 2a). As a result, the removal of water generates empty space in the interlayer for the lower hydration state, which enables less impaired relocation of the intercalated NO_3_^–^ across the *xy*-plane. There is no increase of the self-diffusion coefficient of the intercalated Cl^–^ ion  when the amount of water is lower than for the stable hydration state containing two water molecules (Figure 6b). A plateau of  appears, similar to the hydration energy plateau shown in Figure 2b. Overall, the change of  is more significant compared to  with respect to the hydration state, and it can be attributed to the size difference of these two monovalent anions (the size of Cl^–^ (172 pm) is smaller than NO_3_^–^ (179 pm)). The self-diffusion coefficient of the intercalated CO_3_^2–^ is close to zero when the amount of intercalated water decreases to three water molecules per unit cell, as shown in the inlay of Figure 6c. There is no obvious decrease of  after the amount of intercalated water is lower than three water molecules per unit cell. Similar to , the self-diffusion coefficient of C_2_O_4_^2–^ increases slightly when the number of intercalated water molecules per unit cell is one lower than for the stable hydration state, as shown in the inlay of Figure 6d. However, it is noteworthy that  is overall comparably low (similar to ). The relatively low self-diffusion coefficients  and  presumably arise from the fact that both the CO_3_^2–^ and C_2_O_4_^2–^ ions are capable of forming multiple ionic bonds, thus resulting in a stronger interaction with the metal hydroxide layer in comparison to the other two investigated ions. In conclusion, the self-diffusion coefficient was found to be highly dependent on the hydration state and anion type.

Lower *D* values for the intercalated anions indicate that the LDH host–guest system is highly stable. The self-diffusion coefficients of the four anions at the stable hydration state decrease in the order NO_3_^–^ > Cl^–^ > CO_3_^2–^, and C_2_O_4_^2–^. Concomitantly, the stability order obtained from our calculations is in good agreement with affinity orders from the literature: NO_3_^–^ < Cl^–^ < CO_3_^2–^. For CO_3_^2–^ and C_2_O_4_^2–^, the self-diffusion coefficients at the stable hydration state are equivalent. The intercalated CO_3_^2–^ and C_2_O_4_^2–^ ions possess a lower *D* value compared to the intercalated NO_3_^–^ and Cl^–^ anions studied in this work, which indicates that both the LDH-CO_3_^2–^ and LDH-C_2_O_4_^2–^ systems have more stable structures and simultaneously exhibit lower anion-exchange capacity.

**Figure 7:**
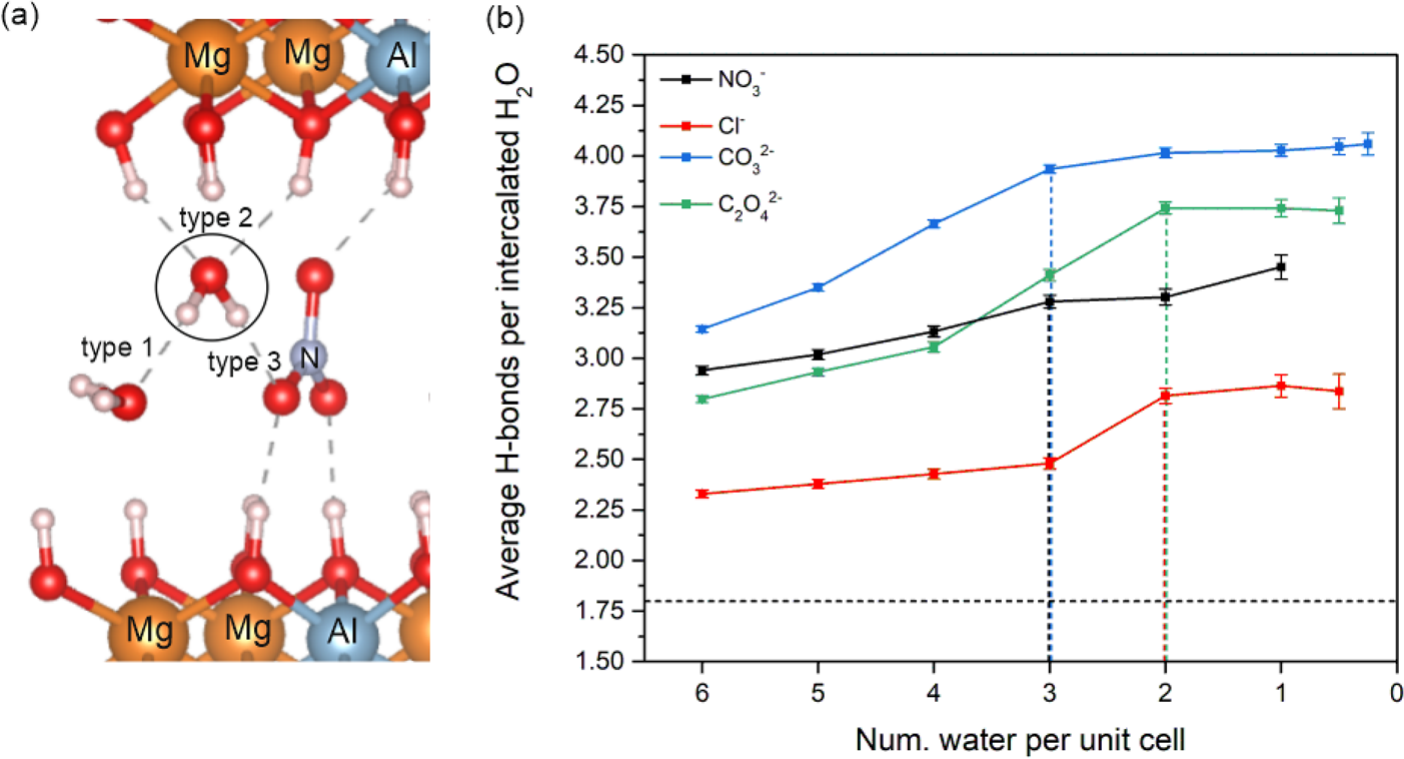
(a) Schematic representation of a hydrogen-bonded network formed around an intercalated water molecule in an LDH-NO_3_^–^ system. Three types of hydrogen bonds are considered: hydrogen bonds formed between water molecules (type 1), hydrogen bonds formed between water and OH groups of the metal hydroxide layer (type 2), and hydrogen bonds formed between water and intercalated anions (type 3). Average hydrogen bonds (H-bonds) per intercalated water was obtained by dividing the sum of the three types of hydrogen bonds by the total number of intercalated water. (b) Average H-bonds per intercalated water at different hydration states for LDH-NO_3_^–^ (black), LDH-Cl^–^ (red), LDH-CO_3_^2–^ (blue), and LDH-C_2_O_4_^2–^ (green). The dotted vertical lines highlight the beginning state of the plateau for each of the investigated LDH systems. The dotted horizontal line stands for the number of hydrogen bonds per water of bulk water obtained from MD calculations.

The hydrogen-bonded network of each LDH system was investigated at different hydration states, since Sasai et al. concluded that this network is important for the stability of the LDH structure.^48^ The average number of hydrogen bonds per intercalated water was derived by dividing the sum of the three types of hydrogen bonds (as shown in Figure 7a) by the total number of intercalated water molecules. Hydrogen bonds formed between intercalated anions and OH groups of the metal hydroxide layer were excluded from the calculation as these were not directly connected to the intercalated water molecules. As shown in Figure 7b, the average number of hydrogen bonds per water molecule typically increases at the beginning of the dehydration process and then reaches a plateau for all four systems. A possible reason for the formation of this plateau could be the reorganization of the intercalated water molecules to maintain the hydrogen-bonded network for the stability. The plateau commences at two water per unit cell for LDH-C_2_O_4_^2–^ and LDH-Cl^–^ and at three water per unit cell for LDH-CO_3_^2–^ and LDH-NO_3_^–^. For LDH-NO_3_^–^, LDH-Cl^–^, and LDH-C_2_O_4_^2–^, the beginning of the plateau correlates with the stable hydration state described in Figure 2a,b,d. For the LDH-CO_3_^2–^, the beginning of its plateau is at a higher hydration state compared to the stable hydration state. The interlayer distance of LDH-CO_3_^2–^ does not significantly change from the beginning state of the plateau to its stable hydration state (see Figure 2c), and all intercalated CO_3_^2–^ are parallel to the metal hydroxide layer as shown in Figure S4. This implies that LDH-CO_3_^2–^ is stable at all these hydration states, since the removal of a water molecule does not destabilize the existing hydrogen-bonded network. The LDH-Cl^−^ system formed the lowest average number of hydrogen bonds per water molecule, as shown in Figure 7b. This could be attributed to the fact that the Cl^−^ provides a lower number of acceptors in the calculation of the hydrogen bonds compared to the other three intercalated anions. At the plateaus of the four systems, the LDH-CO_3_^2–^ and LDH-C_2_O_4_^2–^ formed more hydrogen bonds compared to the LDH-NO_3_^–^ and LDH-Cl^–^, which supports the observation that these two systems have more stable structures. Therefore, it is likely that the intercalated C_2_O_4_^2–^ ion behaves similarly to the intercalated CO_3_^2–^, which cannot be easily replaced by NO_3_^–^ or Cl^–^ via an ion-exchange mechanism.

## Conclusions

In this work, the influence of the water content on the structure of layered double hydroxide (LDH) systems has been investigated for four intercalated anions (NO_3_^–^, Cl^–^, CO_3_^2–^, and C_2_O_4_^2–^). The stable hydration state was identified for each host–guest system by employing a combination of density functional theory and molecular dynamic simulations. The favorable number of water molecules in the intergallery of the respective LDH was determined as three for the NO_3_^–^, as two for the Cl^–^ and the C_2_O_4_^2–^ systems, and as half for the CO_3_^2–^ in each unit cell. It is noteworthy that the interlayer distances of LDH-NO_3_^–^, LDH-Cl^–^, and LDH-CO_3_^2–^ at the stable hydration state are close to their respective experimental reference values. Hence, we are confident that the theoretically determined value for the oxalate ion is a good estimate for the future experimental investigation. The newly parametrized force field, which was presented in this work, forms a robust basis for the investigation of additional anions intercalated in LDH.

The atom distribution, radial distribution, orientation, self-diffusion of the intercalated anions, and hydrogen-bonded network connected to the intercalated water molecules have been discussed to provide deeper insights into the interlayer region and to unravel the influence of different hydration states on the properties of the respective LDH systems. The simulation results showed that the behavior of the intercalated C_2_O_4_^2–^ anion resembles intercalated CO_3_^2–^. Hence, it is not surprising that the two systems exhibit similar properties like interlayer distances, orientation of the intercalated species, and low self-diffusion coefficients at their stable hydration states. The theoretical investigation indicates that the LDH-C_2_O_4_^2–^ is a highly stable host–guest system analogous to LDH-CO_3_^2–^. Moreover, it is known that C_2_O_4_^2–^ may act as a ligand for ions like Cr(VI), resulting in the formation of a Cr(C_2_O_4_)^3–^ complex anion.^55^ It was demonstrated that this complex anion can be subsequently intercalated in the interlayer region of LDH causing significantly larger interlayer distances than one would expect from the simulation using the pure oxalate. To investigate this in more detail, future work will focus on a combination of simulations and experiments to further investigate which form is more favorable for C_2_O_4_^2–^ in the LDH.

In addition to the type of the intercalated anions, the amount of intercalated water will also influence the self-diffusion of the intercalated anions by affecting the configuration of the interlayer region and in turn the anion exchange capacity of LDHs. For the intercalated anions, a high self-diffusion coefficient can assist them in being able to be released from the LDH and thereby further protect Al/Mg alloys from corrosion. As our simulations indicate that the capacity to undergo an ion-exchange mechanism is highly dependent on the thermodynamic stability of the host–guest system, we assume that an uptake of additional water in the intergallery will boost the anion-exchange capacity of LDH-based systems. Experimental studies, e.g., X-ray diffraction (XRD), thermogravimetric analysis (TGA), and anion exchange analysis, will be performed in our research group to investigate this aspect in more detail. Therefore, the rational design of LDH-based systems used in corrosion protection does not only require the selection of potent inhibitors but also needs to take the hydration state into account. It is noteworthy that we focused on the intact LDHs in this work so that the edge effect from the LDHs and environmental influences are not included in this study. Toward this end, we are currently performing Grand Canonical Monte Carlo (GCMC) simulations to elucidate the impact of entropic effects on the predominant hydration state in the interlayer region. The LDH-specific force field presented in this work combined with the proposed Grand-Canical-Monte-Carlo approaches would allow the exchange and intercalation of water and ions to be studied more accurately in the future.

## Methods

### Hydration States

We studied Mg_4_Al_2_-LDH intercalated with NO_3_^–^, Cl^–^, CO_3_^2–^, and C_2_O_4_^2–^ with a varying amount of intercalated water in a dehydration process. The ratio of Mg^2+^/Al^3+^ was set to two since it is one of the commonly found LDHs in experiments.^15,25^ The hydration states can be controlled by changing the number of intercalated water molecules. Initially, each unit cell included six water molecules (Mg_4_Al_2_(OH)_12_–2NO_3_–6H_2_O, Mg_4_Al_2_(OH)_12_–2Cl–6H_2_O, Mg_4_Al_2_(OH)_12_–CO_3_–6H_2_O, and Mg_4_Al_2_(OH)_12_–C_2_O_4_–6H_2_O). At each dehydration step, one water molecule per unit cell was removed from the system until there was no water molecule left. We studied two extra hydration states where the number of water molecules per unit cell was between one and zero, corresponding to a half and a quarter water per unit cell. These two states were obtained by deleting a half and three-quarters of the total amount of intercalated water from the state with one water per unit cell, respectively. The geometry optimization of each unit cell was conducted for each dehydration step by employing DFT calculations using the plane-wave (PW) DFT code Vienna Ab Initio Simulation Package (VASP)^56−59^ with the projector-augmented wave (PAW) method.^60,61^ The exchange-correlation (XC) function optB88-vdW^62−67^ was applied in all DFT simulations to take the van der Waals (vdW) interactions into account. A 3 × 3 × 1 Γ-centered grid of *k*-points^68^ was applied to sample the first Brillouin zone.^15^ A cutoff energy of 520 eV for the plane-wave expansion and an electronic convergence criterion of 1e-6 eV were applied. The density-derived electrostatic and chemical (DDEC6) charges were calculated using the Chargemol program^69,70^ based on the charge densities obtained from the DFT calculations. These charges were calculated at different hydration states for each type of atom. The averaged value of the DDEC6 charges among different hydration states was used for the subsequent MD simulations.

The optimized unit cell with six water molecules obtained from DFT was replicated 5 × 10 × 5 for LDH-NO_3_^–^ and LDH-Cl^–^ and 10 × 5 × 5 for LDH-CO_3_^2–^ and LDH-C_2_O_4_^2–^ to obtain a larger system as input for the MD simulations. Periodic boundary conditions were applied in all MD simulations to avoid finite size effects. All presented MD simulations were carried out with the GROMACS 5.1.5 package^71^ using the leapfrog algorithm^72^ for the integration of the equations of motion. The total potential energy in MD is a sum of nonbonded interactions like Lennard-Jones (LJ) and electrostatic interactions and bonded interactions like bond stretching, angle bending, and dihedral torsion. There is a cutoff of 1.4 nm for the nonbonded interactions, with a potential force-switch modifier function for LJ and a combination of the Particle Mesh Ewald method^73^ and the Coulomb potential-shift function for the long-range electrostatic interactions. More details on how to apply these potential switch methods can be found in the work of Perez-Sanchez et al.^15^ The LJ parameters for the metal hydroxide layers were taken from the CLAYFF force field.^74^ The SPC/E model^47^ was selected for the intercalated water. The required parameters of the anions were adopted from work of Cadena et al.^75^ for NO_3_^–^, from Smith et al.^76^ for Cl^–^, and from Schmid et al.^77^ for CO_3_^2–^. To obtain the parameters for C_2_O_4_^2–^, the Automated Topology Builder repository^78^ based on the GROMOS force field^77^ was utilized.

For the MD simulations, each system underwent a stepwise optimization starting with an energy minimization step. Subsequently, simulation steps in the canonical ensemble (*NVT*) and isothermal–isobaric (*NPT*) ensemble were conducted to reach the equilibrium state, where *N* is the number of the atoms, *V* is the volume, *P* is the pressure, and *T* is the temperature of the system. After that, an NPT production run was carried out to obtain the input for the data analysis. The thermostat and barostat used at different steps were the same as in the work of Perez-Sanchez et al.^15^ In the equilibrium steps, the temperature was fixed at 298 K using velocity-rescaling,^79^ and the pressure was fixed at 1 bar with the Berendsen pressure-coupling method.^80^ In the production run, the Nosé–Hoover thermostat^81,82^ and the Parrinello–Rahman barostat^83^ were applied to fix the temperature and pressure. After the production simulation, a fixed amount of water (one water per unit cell) was removed, and the production step was rerun to reach a new equilibrium state for the new system. One water per unit cell was stepwise removed until there was no intercalated water left in the system. In this work, we first considered seven hydration states for each type of Mg_4_Al_2_-LDH, ranging from six to zero water molecules in the intergallery of the LDH. Additionally, the two extra hydration states with the number of water per unit cell below one were carried out for the LDH-Cl^–^, LDH-CO_3_^2–^ and LDH-C_2_O_4_^2–^ systems to assist the definition of their stable hydration, respectively. To obtain the number of hydrogen bonds per water of bulk water, we built a cubic box filled with water molecules and followed the same simulation steps as for the LDH systems to reach equilibrium. The hydrogen bonds between water molecules were calculated based on the output from the production run.

### Analysis

For different Mg_4_Al_2_-LDHs with different hydration states, the structure properties including number density distribution, radial distribution function, orientation of the intercalated anions (angle calculation between anion plane and the metal hydroxide layer), as well as the self-diffusion coefficients of the intercalated anions were obtained from the last 10 ns of the production simulations using the gmx density, gmx rdf, gmx gangle, and gmx msd tools in GROMACS. The total potential energy was obtained by the gmx energy tool. These calculations provide more insights into the influence of the water content on the behavior of LDH systems with intercalated anions. The hydration energy was defined as
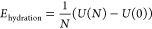
1where *U*(*N*) – *U*(0) is the total potential energy difference between the state with *N* intercalated water molecules and the state without intercalated water.

The interlayer distance of the LDH was estimated by dividing the simulation box size in the *z*-direction by the number of explicitly modeled LDH layers. The number density of any type of atoms was calculated along the *z*-direction to provide the cross-section information on the LDH. In this calculation, the whole system was divided into 500 cells along the *z*-direction, and the number of the atoms was accordingly calculated at each cell and subsequently divided by its volume. As there were five LDH layers in the *z*-direction, we therefore obtained five repeated patterns for the number density distribution. To obtain a good overview of the whole LDH, the five repeated patterns were averaged. To calculate the angle between anion and metal hydroxide layer, the definition of the anion plane for NO_3_^–^, CO_3_^2–^ ,and C_2_O_4_^2–^ is shown in the Supporting Information, with the metal hydroxide layer defined as the *xy*-plane. All angles between the anion plane and the metal hydroxide layer were collected in the last 10 ns. The probability for different angles was calculated to show the angle distribution.

The radial distribution function *g*(*r*) was applied to determine the relative position between intercalated water and anions. Furthermore, the coordination number of water molecules around the anions could be obtained by integration of *g*(*r*) over *r*. After the space around the anions was divided into equally spaced shells, the *g*(*r*) was calculated with the densities of the water molecules in the shells divided by the averaged density of the water in the system, as shown in eq 2 where *B* denotes the intercalated water molecules and *A* the intercalated anions.
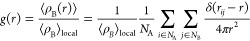
2In the radial distribution function calculation, the center of mass was used for NO_3_^–^, CO_3_^2–^, and C_2_O_4_^2–^ and the oxygen atom for H_2_O. The self-diffusion coefficient was calculated by the Einstein equation,^84^ relating the mean squared displacement to the time
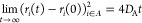
3The mean squared displacement describes the deviation of the anions from their reference positions over time. Analogously to the RDF calculation, the center of mass was used to define the position of NO_3_^–^, CO_3_^2–^ and C_2_O_4_^2–^ and to calculate their displacement. The calculation of the self-diffusion coefficient focused only on the *x*- and *y*-direction since the diffusion in the *z*-direction can be neglected after the system reached equilibrium.

The hydrogen bonds were calculated on the basis of the cutoffs of the angle (hydrogen donor–acceptor) and the distance (donor–acceptor) in gromacs with the command gmx hbond. The cutoff of the angle is ∼30°, and the distance cutoff is 0.35 nm. The OH groups are regarded as donors, and O (and Cl) is an acceptor in this calculation.

## Supporting Information Available

Plots of the DDEC6 charges of all atoms at different hydration states, the definition of the anion planes, the mean squared displacement (MSD) of the intercalated anions, and the angle distribution of the intercalated CO_3_^2–^ with respect to the metal hydroxide layer at different hydration states (PDF)
