# Supplementary material for: Atomistic Insight into the Hydration States of Layered Double Hydroxides
Source: ACS Omega. 2022 Apr 2;7(14):12412–23. doi: 10.1021/acsomega.2c01115 (PMC9016811; doi:10.1021/acsomega.2c01115)
Supplement: Supplementary file 1 — ao2c01115_si_001.pdf [file ao2c01115_si_001.pdf]

Supporting Information: An Atomistic Insight Into the Hydration States of Layered Double Hydroxides

Xuejiao Li,[*,†] Tim Würger,[†,‡] Christian Feiler,[†] Robert H. Meißner,[†,‡] Maria Serdechnova,[†] Carsten Blawert,[†] and Mikhail L. Zheludkevich[*,†,¶]

†*Institute of Surface Science, Helmholtz-Zentrum Hereon, Geesthacht, Germany*

‡*Institute of Polymer and Composites, Hamburg University of Technology, Hamburg, Germany*

¶*Institute for Materials Science, Faculty of Engineering, Kiel University, Kiel, Germany*

E-mail: xuejiao.li@hereon.de; mikhail.zheludkevich@hereon.de

1 DDEC6 partial charges

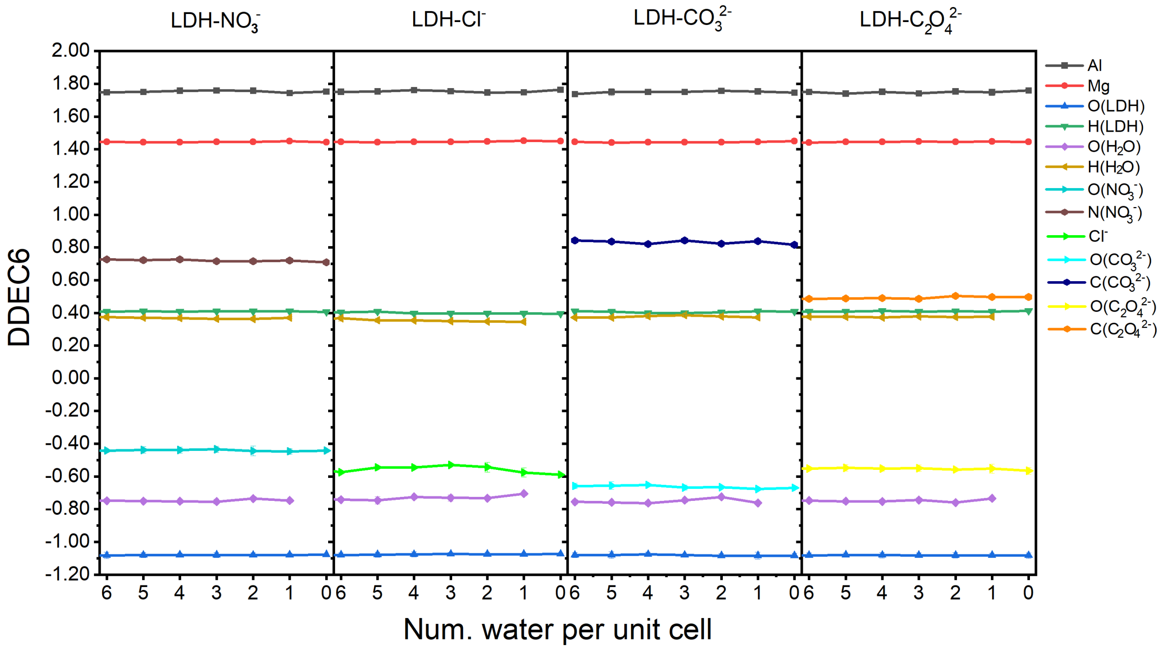

Figure S1: DDEC6 charges of different types of atoms for each of the investigated LDH systems (from left to right are LDH-NO_3^-, LDH-Cl^-, LDH-CO_3^{2-}) and LDH-$C_2O_4^{2-}$) at different hydration states. The color code is shown in the legend and as follows: black for aluminium, red for magnesium, blue for hydroxide oxygen, dark green for hydroxide hydrogen, light purple for water oxygen, dark yellow for water hydrogen, turquoise for nitrate oxygen, brown for nitrate nitrogen, green for chloride, cyan for carbonate oxygen, navy for carbonate carbon, yellow for oxalate oxygen and orange for oxalate carbon.

2 The definition of the planes for the intercalated anions.

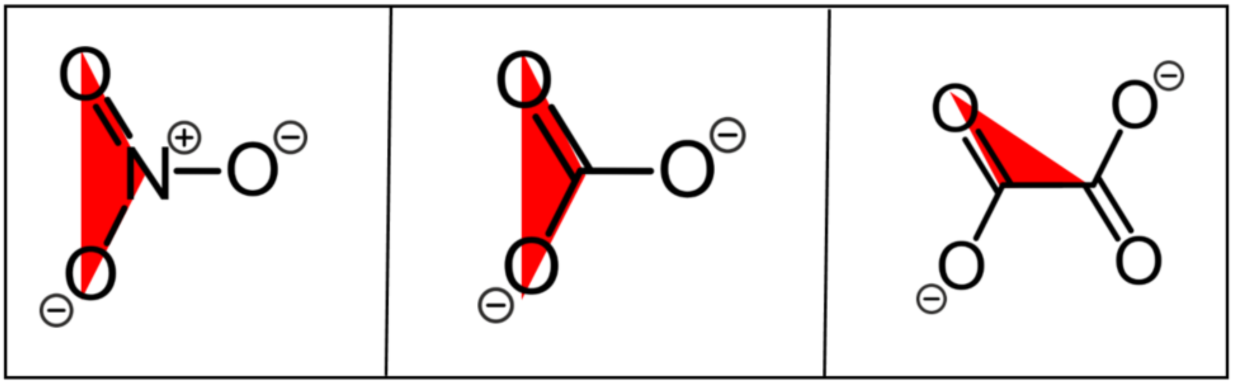

Figure S2: The definition of the planes for the intercalated anions. The anion plane for NO_3^- (left) was defined by one nitrogen and two oxygens forming a triangle as marked in red, for CO_3^{2-} (middle) by one carbon and two oxygens forming a triangle as marked in red and for $C_2O_4^{2-}$ (right) by two carbons and one oxygen atom marked as a triangle in red.

3 Mean squared displacement (MSD) at different hydration states for the intercalated anions.

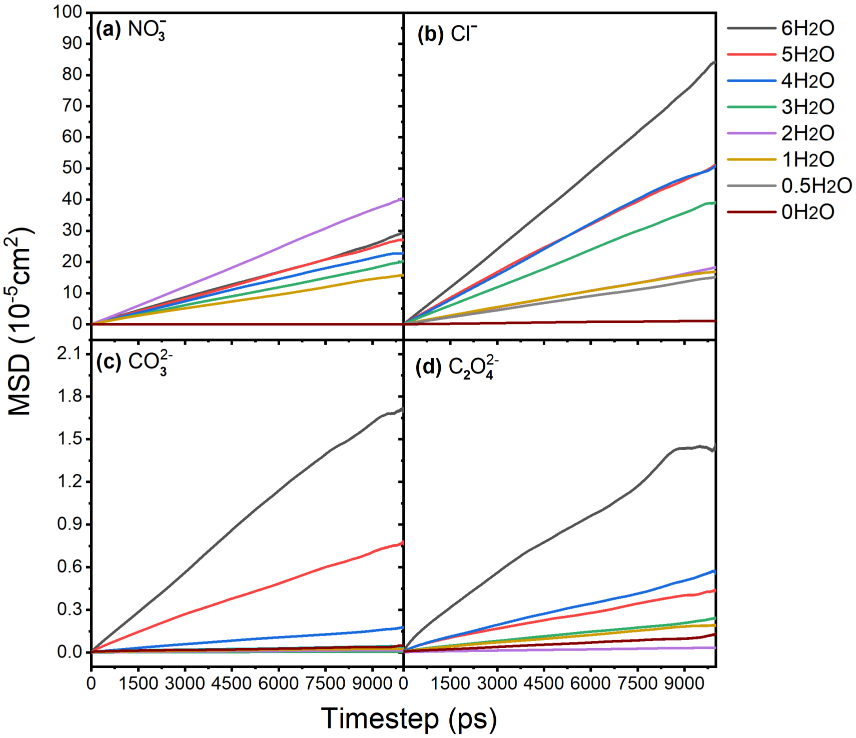

Figure S3: Mean squared displacement (MSD) at different hydration states for (**a**) NO_3^-, (**b**) Cl^-, (**c**) CO_3^{2-} and (**d**) $C_2O_4^{2-}$.

4 Angle distribution of the intercalated CO_3^{2-} with respect to the metal hydroxide layer at different hydration states.

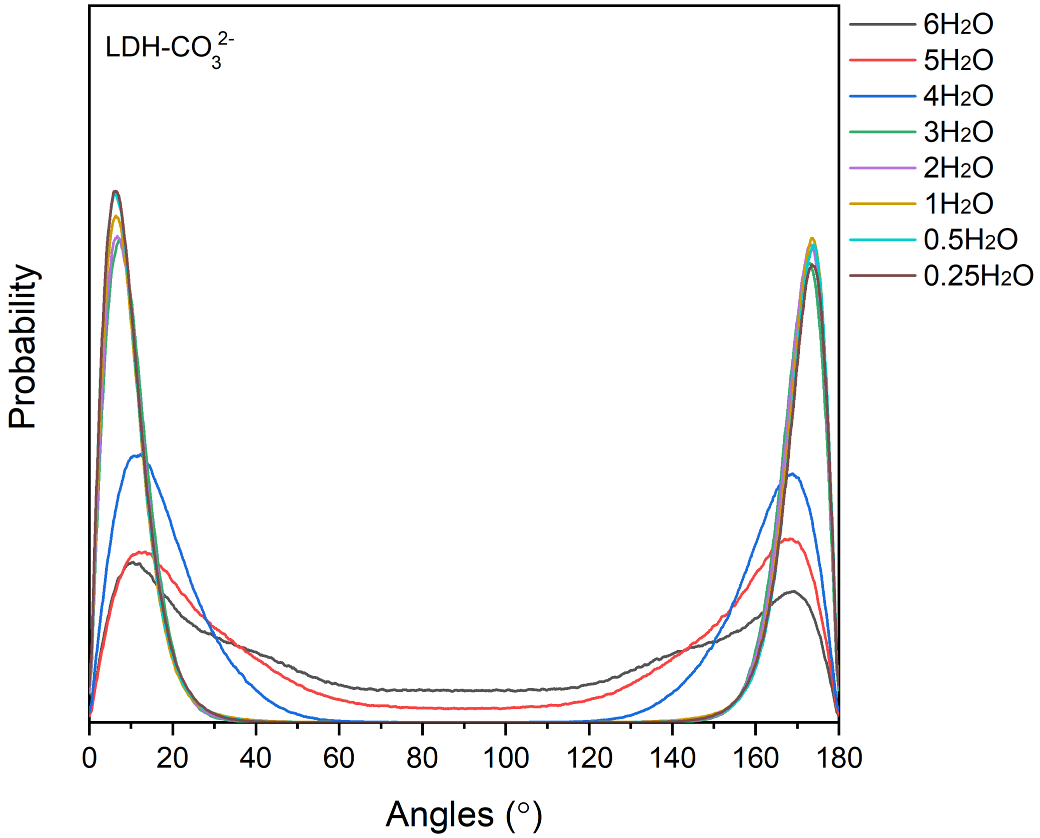

Figure S4: Angle distribution between CO_3^{2-} plane and the metal hydroxide (MOH) layer.
